# Cerebellum and Aging: Update and Challenges

**DOI:** 10.14336/AD.2024.0220

**Published:** 2024-03-01

**Authors:** Christopher L. McElroy, Brian Wang, Hongxia Zhang, Kunlin Jin

**Affiliations:** Department of Pharmacology and Neuroscience, Institute for Healthy Aging, University of North Texas Health Science Center, Fort Worth, TX 76107, USA

**Keywords:** cerebellum, aging, Alzheimer’s disease, Parkinson’s disease, multiple sclerosis, alcoholism

## Abstract

The cerebellum plays a vital role in the aging process. With the aging of the cerebellum, there is a decline in balance and motor function, particularly fine motor skills, and an increased risk of falling. However, in recent years, numerous studies have revealed that the cerebellum has several roles besides balance and fine motor skills, such as cognitive function and memory. It also plays a role in many neurodegenerative diseases. Interestingly, the cerebellum ages more rapidly than other brain regions, including the hippocampus. With increasing studies reporting that the cerebellum has a more prominent and interconnected role in the brain, it is essential to understand why aging affects it more, leading to solutions to help curb the accelerated decline. Here, we summarize the cerebellum’s function and look at how it ages at the cellular, molecular, and functional levels. Additionally, we explore the the effects of alcoholism on the aging cerebellum as well as the role of the cerebellum in diseases such as Alzheimer’s, Parkinson’s, and Multiple Sclerosis.

## Introduction

Worldwide, people are living longer than ever before. As a consequence, neurodegenerative diseases are becoming more prevalent [1]. Among the most critical health problems related to aging is the decline in motor function in the elderly [2]. The motor problems that typically occur in aging include disturbances in gait, balance, and fine motor coordination [2, 3] due to aging-induced cerebellar dysfunction [4-8]. Recent statistics from the U.S. showed that one-fourth of individuals over the age of 65 will fall in a year; every 11 seconds, an older adult is treated in an emergency room for a fall, and every 19 minutes, an elderly person dies from a fall. Falling in the elderly has resulted in over 2.8 million reported injuries treated in emergency rooms each year. In 2013, injury-related death was the third leading cause of poor health among persons aged 65 years and older [9]. This suggests a vital role of the cerebellum in age-related motor decline [10], and falling may be an early sign of functional decline caused by aging. Until recently, most research concerning age-related neurodegenerative diseases has focused on the pathological defects in higher brain regions, the most commonly studied being Alzheimer's disease (AD) and Parkinson's disease (PD) [11]. However, changes in other brain areas, including the cerebellum, occur in many older adults and may contribute to these and other diseases. Further insight into the cerebellum changes that could contribute to the decline of motor and possibly cognitive function may help the development of specific interventions.

### History

Around 335-280 BC, Herophilus recognized that the cerebellum was a distinct part of the brain [12]. Galen later (around 131-200 AD) described the vermis as a wormlike outgrowth. However, the first detailed description of the cerebellum was by Malacarne in 1780. He coined several terms to describe the cerebellum we still use today such as pyramid, tonsil, lingual, and uvula [12, 13]. Determining the function of the cerebellum is ongoing, but early research relied on lesion studies. In 1809, Rolando removed the cerebellum in several species of animals and recorded how this affected voluntary movements but noted that sensation was not affected [12, 14]. Flourens, in 1822, noticed that animals with cerebellar damage moved spontaneously, not smoothly, and lacked coordination. In 1844, he further reported stiff-legged locomotion, and, in birds, there was a retraction of the head when the cerebellum was destroyed [12, 14]. In 1902, Babinsky described dysmetria as a particular pathology of the cerebellum. Dysmetria can be tested by placing one’s finger on their nose with eyes closed. Individuals with cerebellar dysfunction cannot do this accurately [14]. After World War I, Holmes observed slower voluntary arm movement on the side where a gunshot wound damaged the cerebellum [14]. He also described four primary functions of the cerebellum as shown through dysfunction caused by lesions: “1) Postural hypotonia and impairment of certain reactions of the toneless muscles; 2) mild degree of asthenia and fatigability of the muscles; 3) abnormality of rate, regularity, and force of voluntary movements, and 4) failure of certain associated movements [12].”

## Cerebellar Structure and Function

### Gross Structure of the Cerebellum

The cerebellum, raised from the rhombencephalon or hindbrain, is located in the posterior cranial fossa, inferior to the tentorium cerebelli. The cerebellum can be divided into three significant sections depending on the input. The largest section is the cerebrocerebellum and makes up most of the lateral cerebellar hemisphere. It receives its input from several parts of the cerebral cortex and is responsible for complex movements, which include the planning of intricate spatial and temporal sequences, including speech [15, 16]. The vestibulocerebellum is phylogenetically the oldest part of the cerebellum and is composed of the caudal lobes that consist of the flocculus and nodulus. This region receives input from the vestibular nuclei and mainly regulates posture. The spinocerebellum subdivision directly obtains input from the spinal cord. The lateral portion involves distal motor movements, and the middle, the vermis, is involved with eye movements and other close muscle groups [16]. This is only a fundamental breakdown of the cerebellum but is how it has been understood for a long time.

Recently, there have been studies linking the subsections of the cerebellum to non-motor related functions. It was hypothesized that the anterior lobe and lobule VIII represent the sensorimotor part of the cerebellum. Further, lobules VI and VII of the posterior lobes of the cerebellum may contribute to cognition while the posterior vermis is involved in the limbic system. The review of Stoodly and Schmahmann [17] explains this in exhaustive detail.

### The Anatomical Structure of the Cerebellum and its Cellular Makeup

The cerebellum is surprisingly intricate. To better understand its various functions, each layer should be adequately explained. First, the inferior olive, which is not part of the cerebellum but inherently involved, is known to provide the cerebellum with climbing fibers that act as a timing mechanism for movement [18, 19]. These fibers transverse the inferior peduncle to the cerebellum. The inferior peduncle consists of the dorsospinocerebellar tract (DSCT), the cuneocerebellar tract (CCT), and the vestibulocerebellar tract (VCT). The inferior olive is the sole source of climbing fibers that are excitatory synapses on Purkinje cells; it also synapses to the deep cerebellar nuclei. The DSCT, CCT, VCT, and pontocerebellar tract (PCT) axons are mossy fibers that pass through the cerebellar white matter and are excitatory synapses onto the granule cells in the cerebellar cortex. In turn, the granule cells have parallel fibers that run laterally in the cerebellar cortex and have weak excitatory synapses onto the dendrites of the Purkinje cells, which, with sufficient synapses, will cause an action potential and a simple spike rather than the climbing fibers, which causes a complex spike. There are also inhibitory cells in the cerebellum's cortex; Golgi cells and basket cells are excited by parallel fibers. The Golgi cell inhibits the granule cell dendrites, called feedback inhibition. The basket cells' axons terminate and inhibit the Purkinje cells' soma directly, called feedforward inhibition [20]. There are also stellate cells; aspiny stellate cells are inhibitory, and spiny stellate cells are excitatory onto the dendrites of the Purkinje cells [21]. Finally, the Purkinje cells project to the deep cerebellar nuclei, different nuclei for different zones. The lateral zone Purkinje neurons project to the dentate. The interpositus gets its projections from the intermediate zone. The fastigial nucleus gets its projections from the medial zone. The middle cerebellar peduncle contains the PCT. The efferent fibers of the cerebellum, in the superior peduncle, are derived from the cerebellar nuclei and connect to the thalamus [20]. Lastly, the floculonodular zone projects to the “surrogate” deep cerebellar nucleus called the vestibular nuclei [20]. This review will focus on how aging affects each cellular component (Purkinje cells, stellate cells, Golgi cells, and basket cells).

### Interconnections Between the Cerebellum and the Cerebrum

Another critical aspect of the cerebellum is its interconnections with other brain parts. The cerebellum was thought to act as a funnel for cortical areas into the motor system. Still, the cerebellum has projections to many of the same cortical regions that send projections to it [22]. Middleton and Strick [22] injected herpes simplex virus type 1 (HSV-1) into the arm portion of the motor cortex (M1) in primates and resulted in the labeling of the interpositus and the dentate of the cerebellum five days later. A similar experiment was performed on the ventral premotor and supplementary areas, and the authors concluded that the cerebellum, particularly the dentate, also has projections toward these areas. Injecting HSV-1 into the Frontal Eye Field (FEF), the prefrontal cortex, and the cerebellar thalamocortical pathway, which is part of Walker’s area involved in working memory, all showed connections to the dentate of the cerebellum [22, 23].

The cerebellum has also been found to mediate non-motor functions such as working memory and tactile sensation through its interconnections. A functional MRI (fMRI) study revealed that superior cerebellar activation was involved in speech articulation while inferior cerebellar activation would be for phonological storage. This could signal a feedforward command to the frontal lobe, completing a phonological loop [24-26]. The cerebellum also has efferent and afferent projections directly to and from the lateral and posterior parts of the hypothalamus. Another non-motor function of the cerebellum is tactile sensation as the cerebellum has several projections to the red nucleus [27, 28]. A study found that where there was contralateral coactivation of the right red nucleus with the left dentate nucleus of the cerebellum, this activation was much more significant with tactile discrimination than simply moving a finger [29]. In lieu of all these connections, the cerebellum's most exciting and recently studied interaction and connection is between it and the prefrontal cortex. This is important because the aging cerebellum could contribute to deficits in cognition.

### Cognitive Theories of the Cerebellum

Schmahmann and Pandya [30] injected tritiated amino acids proline and leucine, which moved retrogradely to the prefrontal cortex's lateral medial and ventral parts. Using an autoradiographic technique, they found that most of the pontine efferents were from the dorsolateral and medial prefrontal cortex, with fewer efferents in the ventrolateral portion. This is important because the feedforward pathway of the cerebrocerebellar system passes through the corticopontine path [30]. These parts of the prefrontal cortex have implications for higher functioning. The prefrontal cortex is separated into different functional regions. The dorsolateral and medial portions are associated with kinesthetics, motivation, and spatial memory. The inferior prefrontal and orbital areas are responsible for emotional and autonomic response inhibition, stimulus significance, object recognition, and memory [30, 31]. In 1996, Schmahmann proposed five rules governing the relationship between the cerebellum and cognitive thought: (1) Incorporating the limbic and associative regions in the cerebrocerebellar circuit is part of the cerebellar contribution to cognitive thinking and emotions; (2) The cerebellum's topographic organization is related to cognition and behavior [32]; (3) Many afferents to the cerebellum from cerebral associative areas help the cerebellum to regulate supramodal functions; (4) The cerebellum contributes to cognition through modulation; (5) The cerebellum’s calculations for the sensorimotor movement are the same as for the associative and paralimbic functions [32, 33]. The interconnections with the cerebellum and other parts of the brain, along with the well-known motor learning and movement aspects, hence making the cerebellum a noteworthy anatomical feature in the aging process.

### Functions of the Cerebellum

Specific important observations reflect the cerebellum's most basic functions, including posture, smooth voluntary movement, and gait.

Posture is an important aspect that includes muscle tone. Holmes had described through examining individuals with cerebellar lesions as having “postural hypotonia,” or decreased muscle tone [12]. A particular study looked at five groups of individuals; each group had a different cerebellar lesion or affliction. The individuals that had damage to the vermis, the spino-cerebellar afferents, or vestibulocerebellum showed that they had a higher sway and improper balance when asked to stand still with their arms crossed and feet 4 cm apart. This was seen when asked to close their eyes rather than have them open, except for the vestibulocerebellar lesioned individuals, which showed that vision did not stabilize balance like the others. Additionally, there was no difference regarding the balance between individuals with damage to the cerebellar hemispheres and normal individuals [34].

Smooth voluntary movement is also attributed to the cerebellum through the cortical-thalamic-cerebellar pathway. A review by Horne and Butler is an excellent review of some of the past theories of how the cerebellum controls different types of motor movement [35]. Another study emphasizes the cerebral volume, focusing more on fine motor skills rather than the importance of the cerebellum. The smaller the cerebrum through atrophy caused by aging, the worse the spiral drawing skills were in aged individuals [36].

Gait is an important and well-known aspect of the cerebellum; it includes our posture, ability to recognize our limbs in space, and timing. An elementary study by Stolze et al. [37] compared healthy individuals with moderate to mild cerebellar damage. Two simple tests, normal locomotion and tandem gait, were conducted. Both were measured using a three-dimensional infrared movement analysis system. In the tandem gait experiment, individuals were asked to put one foot in front of the other on a red tape line attached to a treadmill. The individuals with cerebellar disease showed a high variability in gait. The intonation was slower for the individuals with cerebellar disease, and they had longer contact with the ground. Step width was also increased in the afflicted patients to try to improve stability.

One of the main functions of the cerebellum found later is motor learning. This is a fantastic aspect of the cerebellum, considering that our declarative memory can easily be outdone by most basic computers these days. Still, the ability to learn and express procedural memories cannot even be matched by the most advanced robots using supercomputers [38]. To gain these procedural memories, there are two types of coding for processes for motor learning. “Rate coding” is the primary information coding device, while “temporal coding” spikes in synapses occur with millisecond accuracy [38-42]. Rate coding can be better explained using the Marr-Albus model, where the climbing fiber provides the strength regulating signal to the parallel threads and finally the Purkinje neurons [39, 41]. Albus was more accurate in his version of the model, which was later proven, where climbing fibers cause long-term depression in Purkinje cells [40, 42].

The model proposed by Albus can be seen in a simple model of the vestibule-ocular reflex (VOR) wherein the eyes moving in the opposite direction of the head automatically stabilize vision in relative movement of the head in space [14]. This is done by the flocculus, which receives primary and secondary signals through mossy fibers and has afferent and efferent signals to and from the flocculus [14]. The VOR is important for many studies testing cerebellar function or dysfunction.

Two significant types of motor learning, supervised and reinforced, are typically studied individually [43]. The field is beginning to understand how these processes are carried out behaviorally and neurally [43]. Error-based learning is a type of supervised learning. A good analogy of this would be shooting a basketball at 12 feet instead of 10 feet. Initially, one would not be very good at this; the motion would be full of errors. These errors are corrected through repetition, and the shot becomes more accurate. In other words, there are deviations to a predicted movement, and these errors are corrected through practice [43-45]. Reinforced learning relies on scalar measures that indicate success or failure. Many real-world movements like walking or taking a swing do not have errors at each point [43]. There is an experimental example of this where a screen covers the hand, and there is a cursor underneath, and when the hand moves straight, the cursor moves slightly to the left. There is a target one must hit by adapting to the cursor movements. One group was given visual feedback on whether they hit the mark but none on their training. The others had feedback on their actions and whether the target was hit i.e., allowing for error-based learning. In the rewarded, reinforced learning group, the question posed was whether or not motor command adaptation would occur without changes in the motor-sensory map [46]. Reinforced learning is spared in individuals with cerebellar damage; even in healthy adults, the retention rate of learning is better in reinforced learning than in error-based learning [43].

Motor learning works by including the contralateral primary motor and premotor cortical areas to the side of the movement, and with an increased number of neurons involved, along with the ipsilateral cerebellum, can increase “motor acuity.” This is defined as the speed swap for accuracy in a task [47]. This increase in neuron recruitment can increase the signal-to-noise ratio and, in turn, improve feedback corrections. This is a type of motor skill learning, not an adaptation. As mentioned above, motor skill learning is swapping speed and accuracy without perturbation [47].

## Cerebellar Aging

### How Aging Affects the Gross Structures of the Cerebellum

Although the mechanism underlying cerebellar dysfunction is unclear, compelling evidence indicate that when the cerebellum ages, it starts to shrink in volume as a whole around the mid-fifties for humans, and importantly, it shows earlier senescence than the hippocampus [46-49]. Total cerebellar volume declines with age, as do global cerebellar white matter volume, mean volume of the Purkinje cell body, and region-specific volumes [48]. Volumetric magnetic resonance imaging (MRI) studies show significant volume loss in the cerebellum with age, especially in the vermis that controls bodily posture and locomotion [49, 50]. However, other regions may show atrophy as well [51]. For example, lobules VI, Crus I, Crus II, and VIIb are significantly affected. It does not significantly affect cognitive decline when controlling for the prefrontal cortex [52-54]. Another study concluded that there was a correlation between gray matter of the cerebellum’s vermis and cognition in men but not in women [55]. In addition, a smaller cerebellar volume correlated with a decline in processing speed, memory, and visual reproduction [56]. Looking particularly at the lobules, aging affects the anterior lobules and Crus I most significantly [53]. In one study, the medial hemispheres were not significantly affected by age except for the inferior portion. The lateral parts of the cerebellum were not affected at all [49]. This is in contrast to the findings of Raz et al. [57], where the cerebellar hemispheres aged and shrank faster than expected. Also, looking at the white matter using Diffusion Tensor Imaging (DTI), it was suggested that the degraded white case involving the frontocerebellar and parietocerebellar circuitry could damage the interaction between these brain regions and might correlate with a decline in cognitive function [58].

Procedural learning and non-verbal working memory are associated with reduced volume of the cerebellar hemispheres [59]. It is known that eye-blink conditioning is also a form of implicit learning that may be attributed to the cerebellum [60]. In one study, a tone is signaled followed by a puff of air to the eye. After a time, there is an anticipatory eye-blink to the tone. There are fewer anticipatory eye blinks in older individuals than there are in younger individuals. The older individuals show a correlation between the smaller cerebellar volume and having a weaker link between the tone and the air puff, considering anticipatory blinks [61, 62].

### How Aging Affects the Cerebellar Cellular Components

Aging affects parts of the brain differently. Some regions, like the cerebellum, have significant neuron loss, while other brain regions, like the primary motor cortex, primary visual cortex, prefrontal cortex, hippocampus, and entorhinal cortex, do not [63]. Brain atrophy has been suggested to result partially from the loss of neurons [64]. Indeed, Purkinje cells (PC) appear somewhat sensitive to aging, exhibiting significant changes in morphology and function during senescence. Woodruff-Pak *et al.* [62] used unbiased stereology to estimate the total number of PCs in the cerebellum and pyramidal neurons in the hippocampus. The results revealed a significant loss of PCs but stable pyramidal neurons during brain aging. Importantly, aging-related loss of PCs occurs predominantly in the vermis, a functional region [65, 66], suggesting that these deteriorative changes are associated with age-related motor decline. Relevant to the cerebellum, the inferior olive does not significantly lose neurons with age [65]. There is also no significant age difference in pontine or cerebellar white matter, according to a DTI study [58]. More research needs to be done on the synaptic connections of mossy fibers and climbing fibers, but the research thus far suggest that they are not affected nor do they contribute to the aging process. A stereological study reported that the anterior lobe of the cerebellum was the most affected by age, with a 40.6% loss of neurons and a 40.9% loss of Purkinje cells in the same area. The overall loss of granule cells in the entire cerebellum was 12.7% and 11.7% for Purkinje cells. Contrary to the DTI study, there was a decrease in white matter (26%) with aging [48]. Another stereological study by Tang et al. [67] showed no significant difference in total white matter volume. Still, there were substantial differences in the total length and diameter of the myelinated fibers; the diameter increased with age. Using mice age up to 31 months, a 30% Purkinje cell loss was observed, and only the pontocerebellum showed a significant loss of granule cells [68]. Although true, the parallel fibers lose up to 79% varicosity in the F344 rat cerebella [69]. Few studies address the connections between climbing fibers and their role in aging. At least in an aged mouse model, the Golgi II neurons do not change in number in any part of the cerebellum [70]. Some studies examined the number of basket cells, which tend to decrease with age. One report, in particular, investigated the changes within the basket cells with age. It was shown that there was an increase in the volume of Golgi, dense bodies, and ground substance and a significant decrease in rough endoplasmic reticulum surface area, suggesting a reduction in protein synthesis [70, 71]. This study was essential because it pointed out that there might be differences within other cells apart from commonly measured parameters like cell number and synapses that could affect function.

Purkinje cells are the most vulnerable to aging [48, 72-74]. This can be attributed to them being the sole output of the cerebellum [66] and importantly, they are involved in motor skills, motor learning [75, 76], and even intellect [77]. Older studies studying rats argue that there is no significant difference in Purkinje cell loss, but more recently, a substantial loss of Purkinje neurons, at least in the anterior lobe, has been reported [78, 79]. Many circumstances can cause age-related loss, including impaired L-glutamate catabolizing mechanisms in the cerebellar cortex of rats [80], loss of neuroglobin [81], and even the loss of interaction with parallel fibers [69].

The soma of Purkinje cells can diminish by up to 33% in the human cerebellum [48]. There is also restructuring of the dendritic tree of Purkinje neurons [82-84]. This decrease, particularly in the terminal segments of the dendritic tree, reduces the exchange of synaptic information, which may contribute to the loss of the molecular layer in the aged cerebellum [66, 84]. The reason for this is not well understood. It is supposed that the parallel fibers from the granule cells decrease their inputs on the Purkinje neurons [69, 85, 86]. It has been reported that even organelles change within aged Purkinje neurons. One, in particular, is the mitochondria, which decrease in number with age and could affect cellular function [87]. Succinic dehydrogenase and the volume and number of mitochondria are reduced in aged mitochondria [88]. The nucleus becomes pyknotic, and the nucleolus is also reduced and disrupts Cajal bodies, which decreases the volume in the aged cerebellum [89, 90]. These deficits in the organelles may contribute to cellular death and dysfunction. Synaptic plasticity is another factor that could cause Purkinje cell death. A 33% reduction in Purkinje cell dendritic spines was seen in some older studies [69, 91].

### Functional Deficits Attributed to Aging

By 2030, 1 in 5 U.S. residents will be over the age of 65, and these individuals will face a decline in sensorimotor control [8]. In aged individuals, there is difficulty in coordination [92], loss of fine motor control [93], slowing of choice reaction time [94], as well as balance and gait issues [95]. A simple explanation for early cell death in the cerebellum, particularly in the anterior lobe, is that it disrupts the ability to predict outside and inherent forces acting on the body [96]. In part, the issues with movement in aging are due to the periphery such as sensory receptors, muscles, nerves, and joints [8]. Several unimanual and bimanual tasks were done on 217 adults to evaluate the relationship between cerebellar morphology, age, and manual motor performance, considering cerebellar white and gray matter volume. This study observed a correlation between gray matter and white matter volume with dexterity, grip strength, and finger-tapping performance, which declined with the minor white tissues and gray matter volumes [97].

The cerebellum does not act on its own when performing motor movements. There are two projected motor movement models. The forward model control system imitates the dynamic controlled object (CO) properties in which the output brings the error signals and is filtered through the sensitivity system. It then goes to the inferior olive to the forward model of the cerebellum, where the signal is modified. There is also the inverse model, which has the feedback of the error signals coming directly from the command signals derived from the motor or prefrontal cortex [98]. It was suggested that, in advanced age, forward models would decline mainly, although the inverse model may also be damaged [60].

## Possible Underlying Mechanisms Contributing to Cerebellar Aging

### Molecular Aging of the Cerebellum

Many molecular mechanisms can affect the aging process of the cerebellum, and there are several exciting recent discoveries. One of these studies refutes the hypothesis that the cerebellum is one part of the brain that deteriorates quickly [99]. Instead of the decreased rate of degeneration around 50, this study looked at individuals above 110 years of age and found that the epigenetic clock, which is an evaluation of DNA methylation through aging, was less in the cerebellum than in other brain regions. In other words, epigenetic age, also called DNAm, strongly correlates with chronological age in brain tissue, and the cerebellar samples showed less DNAm change than non-cerebellar samples [100].

With aging, the cerebellum undergoes several changes in metabolic activity and neurotransmitter synthesis. A proteomic screening and immunohistochemistry study on Ercc1 knock-out mice showed that aging affects the expression of many proteins in the cerebellum, including proteins involved in neurotransmitter reception (i.e., mGluR1, GluRδ2, GABABR1, and GABABR2), signaling molecules (i.e., GlurRδ2, Delphilin, and IP3R1), and receptor-signal transduction scaffolds (i.e., cGK1, PKCγ, Ahrgef33, RGS8, TN-C, and Ppp1r16b) [101].

DNA repair also suffers from senescence. Expression of Topoisomerase IIβ (Topo IIβ), a DNA repair enzyme, decreases in aged individuals. On the other hand, several genes that code for inflammatory cytokines are upregulated in senescence-associated secretory phenotypes and are called senescence-associated genes [102]. It was reported that genes Slit2 and Npy gradually increase during aging and that suppressing these two genes increased the viability of cerebellar granule cells and Topo IIβ upregulation *in vitro* [103].

Further, presenilins are crucial proteins in the aging process. The mutation in presenilins can lead to Alzheimer’s disease, and the differential expression of presenilins in normal aging in the cerebellum contributes to deficits in motor coordination during “healthy” aging [104]. This could be a potential therapeutic target. However, healthy aging involves more than a couple of differentially expressed proteins.

An additional study looked at Lipins-1 and -2; they are involved in the synthesis of phosphatidate phosphatase, triglycerides, and phosphatidyl-ethanolamine. Lipin-2, with concomitant reduction of lipin-1 with age, can cause ataxia in Lipin-2 knockout mice [105]. One exciting approach to elucidating the aging cerebellum's molecular profile is Population Specific Expression Analysis (PSEA), which is very useful in heterogeneous samples because it uses genetic profiles to separate cell populations. Using this technique, one study found that the astrocyte population showed differential expression of 50 genes in the aging brain [106]. One reason for this differential expression could be the decrease in the endonuclease Dicer, which is critical for producing miRNAs. Ablation of Dicer in Purkinje cells reduced miRNAs and caused Purkinje cell death and eventual ataxia [107, 108]. These studies demonstrate that genetics is not the only factor affecting the changes caused by aging in the cerebellum.

Sialic acid-containing glycoproteins are also affected. In particular, α2, 8-linked sialic acid (Sia), which can attach to glycoproteins such as NCAM or CD166 via α2, 8-sialyltransferase III (ST8SiaIII), is involved in cell adhesion and neurite growth. diSia epitopes and ST8SiaIII are both reduced in the aging cerebellum of senile mice [109, 110]. This reduction is evident in mice with ataxia and is one consequence of the aging cerebellum. Looking at the cerebrospinal fluid (CSF) of individuals with ataxia, one study revealed that free sialic acid was increased. This could be a possible biomarker for individuals who do not have other obvious issues that could cause ataxia. Unfortunately, sialic acid is only present in the CSF; considering the invasive procedure, it is not the best diagnostic option [111].

There are several ways to protect oneself from the ravages of the aging process. One of these means of protection could be a calorie-restricted diet. This particular study is not necessarily recent, but it shows that we may have some influence on how we age. In particular, the mRNA expression for the α2 subunit of GABAA receptor subunit is significantly decreased in aged rats but not so in calorie-restricted aged rats [112]. Another positive is that Mcl-1 is neuroprotective for cerebellar granule cells. However, this protein is phosphorylated and ubiquitinated with age. This leaves cells more susceptible to DNA damage and apoptosis. There is a therapeutic means of stopping this degradation by inhibiting JNK. This inhibitor is protective against dopaminergic neuron loss and may do the same for cerebellar neurons [113, 114]. Parkin degrades Fbw7β, an SCF substrate adaptor that can ubiquitinate Mcl-1. Increasing Parkin, in turn, increases Mcl-1. This is also a therapeutic target because Parkin can be improved by diaminodiphenyl sulfone, which could help increase neuron survivability [113, 115].

### Vascular Aging of the Cerebellum

In 2019, Prof. Jin put forward a microcirculatory theory of aging, which states that aging is the continuous impairment of microcirculation in the body [116]. Indeed, the most noticeable characteristic of vascular aging is the change in the mechanical and structural properties of the vascular wall, leading to the impairment of vessel functions such as cerebral blood flow (CBF). Importantly, age-related changes in CBF and microvessels appear to be regionally distinct [117-122]. At the same time, vascular decline is one of the significant causes of aging and age-related disease [123]. While various factors may influence the nature and severity of cell degeneration in the cerebellum, the underlying mechanism remains unclear. Notably, the integrity of the vascular system is essential for the efficient functioning of the brain. The macro- or micro-circulation of the brain is susceptible to aging, as is the vascular system of the rest of the body. Aging-related structural and functional disturbances in the macro- or micro-circulation of the brain lead to brain degeneration and dysfunction. Importantly, these significant changes in microvessels of the aging brain are region-specific [124, 125]. Aging also affects the cerebral blood flow, which appears regionally distinct [117]. Several controls fail in the cerebrum, such as autoregulation, neurovascular coupling, BBB leakage, reduced cerebrospinal fluid, and lack of vascular tone, all of which contribute to neurodegeneration [126]. Few reports are available concerning the direct effect of aging on cerebellar blood vessels. One study by Akima et al. [127] compared the vascular aging of the cerebrum to that of the cerebellum. One particular difference in a few advanced aged cerebella was an intertwining or ropelike appearance of arteries in the cerebellum. This was much more pronounced in the cerebrum [127]. Regarding the function of these blood vessels, the blood flow and oxygen consumption through positron emission tomography (PET) scans were studied on young and aged individuals. It was concluded that there was no significant difference in blood flow or oxygen concentration in the cerebellum between young and old individuals [128]. Some older studies have also come to the same conclusion, but no recent work has been done that could dispute their results [129, 130]. This is why this current study is essential. It employs new techniques to reevaluate whether or not any vascular differences could account for some of the physical or functional deficits in the cerebellum.

### Cerebellar Compensations in Aging

Another factor to consider with increased age is how differently the brain, including the cerebellum, participates in motor movements. In aged individuals, the accuracy of pressing the button in a simple button press motor task is not something that declines; the reaction time, however, is affected. The brain compensates by adding more cortical and subcortical regions for simple motor tasks [76]. In a study by Wu and Hallet [131], automaticity was tested between aged and young individuals. In both groups, similar brain areas were activated when learning the directed finger movements and achieving automaticity. Still, aged individuals had a higher activity in the bilateral anterior lobe of the cerebellum, premotor area, parietal cortex, left prefrontal cortex, anterior cingulate, caudate nucleus, and thalamus, and employed more areas comprising the pre-supplementary motor area and the bilateral posterior lobe of the cerebellum [131]. This means that when one ages, simple tasks are not so straightforward anymore. It takes more brain activity to perform the same functions as a young adult, and still, the results still pale in comparison. It would be interesting to determine if this increased compensatory activity creates more errors in coordination between the cerebellum and other brain sections.

## Cerebellar Involvement in Diseases

The cerebellum is unique in how it reacts to different diseases. Fairly recently, more attention has been paid to the cerebellum and its involvement in several other diseases, such as Alzheimer’s, Parkinson’s, multiple sclerosis, and alcoholism.

### Alzheimer’s Disease

In AD, there are amyloid β plaques in the molecular layer of the cerebellum in Braak stage 3 AD patients [132]. For reasons yet unknown, the cerebellum is spared from neurofibrillary tangles. The fact that there are plaques in the cerebellum may perhaps be a symptom of advanced AD, but it is arguable whether there are functional consequences because of this [133]. On a cellular level, there seems to be a loss of Purkinje cells in the AD brain, which can be salvaged through upregulating heat shock factor 1 (HSF1), and thus increase the expression of heat shock proteins 60, 70 and 90 [134]. Synaptically, Purkinje cells are affected by alterations in their intrinsic excitability, and the release of GABA is lowered in interneurons in the cerebellum [135]. Considering the effects on the Purkinje cells, one could conclude that AD in the cerebellum accelerates the aging process. Furthermore, it was revealed through MRI that AD and frontal temporal dementia are connected with focal volume loss in the cerebellum. Therefore, although AD’s effect on motor function in the cerebellum may be debatable, it seems to contribute to inevitable dementia related to the disease [136, 137].

### Parkinson’s Disease

Parkinson’s disease (PD) is described as a progressive decline in motor skills, including gait, posture, rigidity, and resting tremor. Most of the attention has been put on the basal ganglia regarding the pathophysiology and potential treatment for the disease. The reader is directed to Wu and Hallet [138] for an extensive review. This section will focus on the more recent developments involving the cerebellum.

One of the symptoms of the disease is the change in α-synuclein [139, 140]. Although α-synuclein is increased in the basal ganglia, it is arguably reduced in the cerebellum in PD patients [141, 142]. More study needs to be done to clarify the role of α-synuclein in the cerebellum. According to Hurley et al. [143], there are dopamine 1 (D1) and dopamine 3 (D3) receptors in the cerebellum, as well as the presence of tyrosine hydroxylase, which were found in lobules 9 and 10. In PD patients, the mRNA of D1, D3 receptors, and tyrosine hydroxylase are reduced, suggesting that the cerebellum may have an active role in the motor dysfunction in the disease. There are also morphological changes in the cerebellum due to PD. Borghammer et al. [144] found that the left cerebellum was smaller than the average of the age-matched controls using deformation-based morphometry, which is touted to be more sensitive than voxel-based morphometry. The cerebellum also increases in activation in individuals with Parkinson’s based on (blood oxygen level-dependent magnetic resonance imaging) BOLD MRI, and PET studies when performing different tasks that involve motor learning and motor execution [138]. The increased activation is also valid in individuals with akinesia/rigidity Parkinson’s at rest [145]. In another report, rats were subjected to dopaminergic deafferentation via 6-hydroxydopamine lesions and then made to perform skilled or non-skilled aerobic exercise. The skilled aerobic exercised animals had a greater rCBF in the cerebellum and had excellent connectivity between the prelimbic cortex and motor areas. They changed the functional connection between the brain's midline cerebellum and sensorimotor parts, particularly the cerebellar-thalamocortical compensatory circuit [146]. This compensatory reorganization is also seen in individuals who undergo ventral intermediate nucleus thalamotomy [147]. Interestingly, levodopa changes this reorganization to normal [145]. This begs whether the restructuring of the cerebellum is part of the pathological process of Parkinson’s disease or is merely a compensatory mechanism. To answer this question, one must suppress cerebellar activity and observe whether the symptoms improve or worsen in a PD animal model [138].

### Multiple Sclerosis

Another neurodegenerative disease that begins at a younger age is multiple sclerosis (MS). Although the onset of MS starts earlier in life, the average age of the individual with MS has increased dramatically [148]. A study out of British Columbia, which has a high rate of MS, reported that the average age increased from 45-49 in the early 90s to 55-59 years in 2008 [149]. Another study in Genoa, Italy, saw 18% of people who had MS over the age of 65 [150]. Overall, the younger the beginning, the longer the survivability of individuals when switching from exacerbating-remitting inception and the inception of secondary progression, as well as the lower the rate of actually switching to secondary progression MS [148, 151, 152]. In essence, although MS is not necessarily age-dependent like AD or PD, its progression is dependent on age.

Previously, the focus was mainly on the telencephalic brain regions, but the cerebellum has only recently received attention [153, 154]. One study found that there is extensive cortical demyelination of the cerebellum, especially in individuals with progressive MS. It was shown that there was a moderate loss of Purkinje cells but otherwise preservation of most of the neurons and their axons, although the axons showed swelling. Purkinje cell loss was only observed in individuals with leukocortical lesions [155]. In MS, demyelination can be rather severe. Typically, demyelination is about 30% to 40% but can increase to around 90%. Even though one would expect some functional consequences, it is still unclear what the extent of those consequences is [156]. Individuals who demonstrate signs of cerebellar damage tend to have a quicker and more disabling progression of the disease [157].

One relatively unexplored area of MS affecting the cerebellum is how it is involved in cognitive decline. Valentino et al. [158] showed that individuals with cerebellar motor deficits also had deficits in attention and verbal fluency compared with individuals with MS who did not show cerebellar dysfunction. Likewise, a different study came to a similar conclusion but used an anti-saccade task that could assess cognitive function [159]. MS also affects working memory and processing speed [154]. One reason for the motor and cognitive deficits is the inflammation associated with MS. CD4+ and CD8+ T-cells, B cells, macrophages, and plasma cells are involved in this inflammation, which occurs mainly in the meninges and spreads to the subpial cortices [160]. Howell et al. [161] investigated this inflammation in the cerebellum because of its deep-folded sulci. They found that inflammation in the subarachnoid space contributes to greater subpial demyelination in the cerebellum. It has also been reported that the lesions in the cortical and the white matter parts of the cerebellum contribute to an increased Kurtzke expanded disability status scale [162]. Not only are the lesions attributed to cerebellar dysfunction, but there is also a molecular component. IL-1β is an inflammatory cytokine that is highly expressed in mice models of MS, and it reduces GABA-ergic transmission. One possible direction to look at for treatment is blocking IL-1β via IL-1_ra_, a receptor antagonist, and thus alleviate inflammation [163]. An interesting note about MS is that it affects the homogeneity of the left cerebellum, particularly that of Parkinson’s disease. This lack of uniformity could be due to reduced cortico-ponto-cerebellar and spino-cerebellar inputs and could contribute to higher ataxia scores [153]. Incidentally, a study by Shields et al. [164] found that a blocker for the sodium channel Nav1.8, PF-01247324, can be administered per os, which alleviated cerebellar symptoms in an experimental autoimmune encephalomyelitis (EAE) mouse model without noticeable side effects because of its selectivity. Another positive in the research on MS is the discovery of specific miRNA biomarkers that can assess the progression, detection, and remission of the disease and might be a diagnostic in the future [165].

### Alcoholism

Although healthy brain aging is ideal and having a disease such as PD, AD, or MS cannot necessarily be helped, we do have the choice of reducing aging risks by avoiding excessive drinking of alcohol. It is well known that alcoholism can cause ataxia by atrophy of the cerebellum and peripheral neuropathy [166]. Complicating alcoholism is a thiamine deficiency that results in ataxia, confusion, and ophthalmoplegia, called Wernicke’s encephalopathy. Many also suffer from Korsakoff syndrome simultaneously, which entails agitated delirium and delirium tremens; together, it is termed Wernicke-Korsakoff syndrome [167].

Independent of those complications, there are two hypotheses regarding how alcoholism affects the way the brain ages. One hypothesis is the “accelerated aging hypothesis.” This model purports that an individual ages cognitively and possibly psychologically before they are supposed to age, which begins early on in adulthood. The second hypothesis is the “age sensitivity hypothesis.” In this model, once an individual starts showing age-related cognitive changes around their 40’s if they abused alcohol, they would show more significant changes than individuals who did not drink excessively [168, 169]. In a SPECT study by Harris et al. [169], cortical/cerebellar blood perfusion was measured under the assumption of the “age sensitivity hypothesis.” The cerebellum was found to be hypoperfused to a more significant extent in abstinent alcoholics than their paired controls, and the hypoperfusion was more significant in the cerebellum than in the cerebral cortex. This was more pronounced with individuals whose last drink was later in life. Not only is the blood flow impaired in abstinent alcoholics, but there is more significant shrinkage of the cerebellum vermis in alcoholics [170]. There is atrophy in the white matter, too, which is seemingly disproportionate to the other layers of the cerebellum. This may indicate a particular degeneration of axons of the cerebellum [171]. The white matter degeneration in the cerebellar hemispheres was evident in individuals with Korsakoff’s syndrome but not in individuals with uncomplicated alcoholism [172]. In the anterior vermis, gray and white matter were reduced, and the amount of ataxia an individual displayed correlated with the white matter atrophy in this area [172]. GABA_A_ receptor inhibition has increased in Purkinje and granule cells with acute alcohol exposure [173]. It was seen in some early studies that the granule cells and interneurons were vulnerable to atrophy from alcoholism. Still, more recent studies show that there is no significant reduction in number [174-177]. This is only true if the individual does not suffer from Wernicke’s disease, which can induce a 57% loss of Purkinje cells [178]. Even though there may not be an excessive loss of Purkinje cells in alcoholics without Wernicke’s, there is a change in the dendritic arbor. Pentney [179] discovered that when twelve-month-old rats were treated with ethanol for 24 or 48 weeks, there was an elongation of terminal segments of dendrites. A later study concluded that aged ethanol-fed rats had a dilation of smooth endoplasmic reticulum in their dendritic arbors [180]. These could account for the ataxia induced by alcoholism later in life. Alcoholism is considered to be a disease that affects millions of people. Confronting the problem earlier in life will help reduce the effects of the sensitivity induced by alcoholism and the damage caused by aging considerably.

## Challenges

Through the last several decades, the cerebellum has been found to be more than just a structure that controls fine motor movements. The cerebellum has recently garnered more attention, and for good reason. It connects to other brain parts, such as speech, working memory, tactile sensation, and cognition. It may also be one of the brain structures that age the soonest. Given all the implications this may have, the cerebellum warrants further study. It is already established that the aging cerebellum affects balance, gait, posture, and how quickly one performs fine motor movements. Interestingly, aging affects parts of the brain differently. Some regions, like the cerebellum, have significant neuronal loss, while other brain regions, like the primary motor cortex, primary visual cortex, prefrontal cortex, hippocampus, and entorhinal cortex, do not [63]. Relevant to the cerebellum, the inferior olive does not significantly lose neurons with age [65]. There is also no significant age difference in pontine or cerebellar white matter [58]. However, the anterior lobe of the cerebellum was the most affected by age, with a 40.9% loss of Purkinje cells. Taken together, motor deficits, such as falling, the earlier signs of brain aging, and neuronal degeneration in the vermis of the cerebellum may be the leading cause of these signs. Looking forward, how aging affects the cerebellum’s connections with the other parts of the brain would be key in uncovering the crucial roles the cerebellum play in age-related neurological diseases.
